# Retina-Inspired Models Enhance Visual Saliency Prediction

**DOI:** 10.3390/e27040436

**Published:** 2025-04-18

**Authors:** Gang Shen, Wenjun Ma, Wen Zhai, Xuefei Lv, Guangyao Chen, Yonghong Tian

**Affiliations:** 1 Smart Tower Co., Ltd., Beijing 100089, China; shengang3@chinatowercom.cn (G.S.); mawj@chinatowercom.cn (W.M.); lvxf@chinatowercom.cn (X.L.); 2 State Unclear Electric Power Planning Design & Research Institute Co., Ltd., Beijing 100095, China; 3 School of Computer Science, Peking University, Beijing 100191, China; yhtian@pku.edu.cn

**Keywords:** visual saliency, retina imitation, saliency enhancement, entropy reduction, information theory

## Abstract

Biologically inspired retinal preprocessing improves visual perception by efficiently encoding and reducing entropy in images. In this study, we introduce a new saliency prediction framework that combines a retinal model with deep neural networks (DNNs) using information theory ideas. By mimicking the human retina, our method creates clearer saliency maps with lower entropy and supports efficient computation with DNNs by optimizing information flow and reducing redundancy. We treat saliency prediction as an information maximization problem, where important regions have high information and low local entropy. Tests on several benchmark datasets show that adding the retinal model boosts the performance of various bottom-up saliency prediction methods by better managing information and reducing uncertainty. We use metrics like mutual information and entropy to measure improvements in accuracy and efficiency. Our framework outperforms state-of-the-art models, producing saliency maps that closely match where people actually look. By combining neurobiological insights with information theory—using measures like Kullback–Leibler divergence and information gain—our method not only improves prediction accuracy but also offers a clear, quantitative understanding of saliency. This approach shows promise for future research that brings together neuroscience, entropy, and deep learning to enhance visual saliency prediction.

## 1. Introduction

The human visual system (HVS) comprises several hierarchical layers, including the retina, V1, and V2, which collectively initiate the intricate process of visual perception [1]. Among these, the retina serves as the primary interface for natural scene processing, transforming incoming light into neural signals suitable for transmission to the brain. This transformation is not only a biological feat but also an information-theoretic one, as the retina encodes visual information while reducing redundancy and entropy. Retinal processing exhibits numerous fundamental features essential for subsequent cortical interpretation, such as band-pass filtering, gain control, and spiking synchrony [1], all of which contribute to efficient entropy management. Unlike electronic cameras, which often lose detail and color fidelity in low-light conditions or at distances from light sources, the retina’s Parvocellular pathway preserves detailed information and enhances color contrast, thereby guiding human gaze toward salient regions in static scenes [2]. This highlights the retina’s pivotal role in the HVS’s ability to effectively process natural scenes by maximizing relevant information and minimizing extraneous entropy.

The HVS excels at rapidly prioritizing visual stimuli, enabling the selection of salient regions from complex environments. Mimicking this capability in computational models has been a longstanding goal in computer vision research. Visual saliency prediction [3,4,5,6,7,8,9,10] aims to estimate the regions within an image that are most likely to attract human attention. Along the visual pathway, various cortical areas, including the retina, lateral geniculate nucleus (LGN), V1, and higher-level brain regions, collaboratively contribute to the determination of saliency. Consequently, the output of retinal processing is critical for accurate saliency estimation, as it initiates a cascade of information-theoretic transformations that reduce uncertainty and enhance signal-to-noise ratio. In this study, we investigate the impact of retina imitation on saliency prediction through a series of experiments grounded in entropy and information theory.

**Figure 1:**
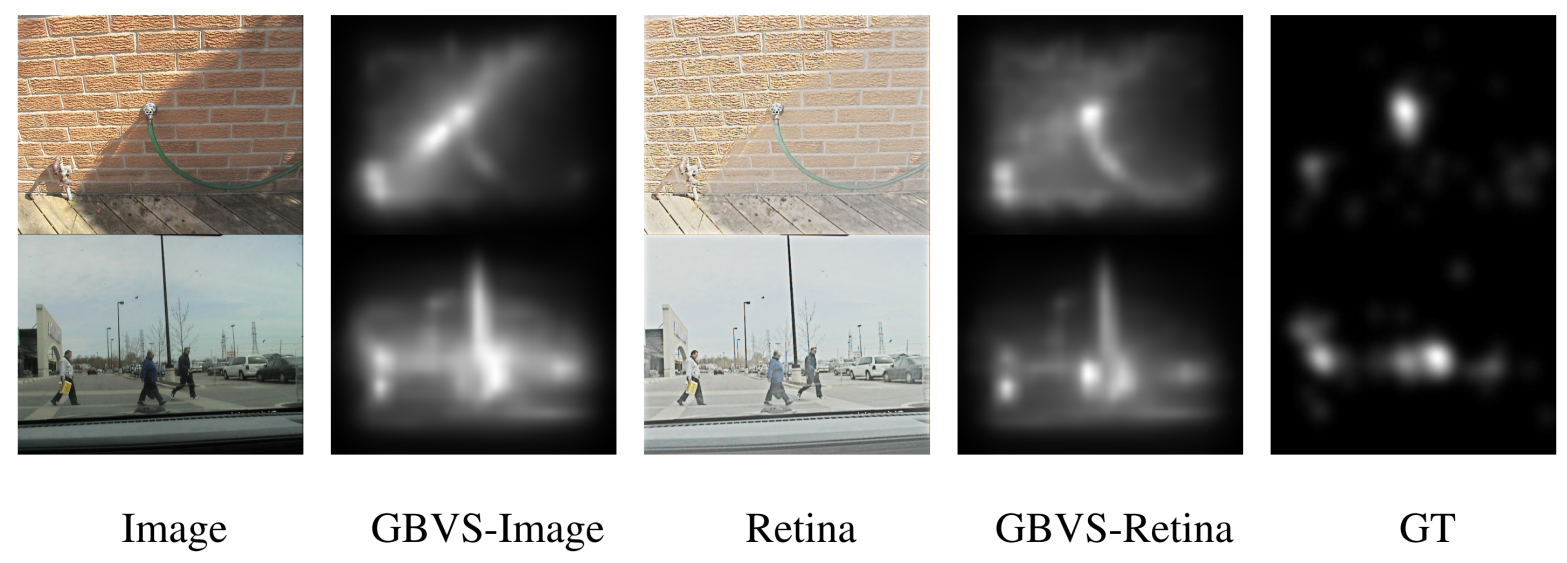
Comparison of different processing methods. Column 1 shows the original street scene with buildings and pedestrians. Column 2 displays the GBVS [14] saliency map, where brighter areas are more salient. Column 3 shows a retina simulation mimicking human vision. Column 4 presents the GBVS saliency map applied to the retina-processed image. Column 5 is the ground truth with human-labeled attention regions. This comparison suggests that retina-based processing better aligns saliency maps with human attention.

Biologically inspired retinal models have been extensively developed, ranging from detailed simulations of specific physiological phenomena to comprehensive models encompassing the entire retina. Early efforts, such as those by Mead and Mahowald [11], sought to emulate the neurophysiological properties of vertebrate retinas by drawing analogies with electronic circuits, implicitly considering the flow and preservation of information. The Virtual Retina model [12] successfully reproduces the biological complexity of retinal processing while maintaining the efficiency of functional models and managing the entropy of the transmitted signals. Building upon Mead’s work, Benoit’s model [13] adopts a global approach to retinal processing, leveraging analogies between electronic circuits and the retina’s signal processing strategies. This model emphasizes the distinct properties of retinal cell networks, natural parallel processing, and information optimization to enable rapid computation and entropy reduction. Given our focus on enhancing image saliency, we adopt aspects of Benoit’s model to augment saliency prediction algorithms, as illustrated in Figure 1.

Parallel advancements in deep learning have revealed striking parallels between neural processing in the brain and deep neural networks (DNNs), particularly in the domain of vision. Recent studies, such as those by Cichy et al. [15], have demonstrated a correspondence between the hierarchical organization of human visual areas and the layered structure of DNNs, suggesting that DNNs effectively model visual object recognition processes akin to those in the primate brain. These models can be interpreted through an information-theoretic lens, where each layer acts to transform and compress visual data while preserving salient features and reducing entropy. Cichy et al. [15] compared temporal and spatial representations in the human visual cortex with those in artificial DNNs trained for real-world visual recognition tasks, revealing that DNNs capture the stages of human visual processing from early visual areas to the dorsal and ventral streams. Building on these insights, our study integrates retinal processing models with DNN architectures to develop a more biologically plausible and effective saliency prediction framework, leveraging entropy and information theory concepts to improve performance and interpretability, as detailed in Section 4.

The remainder of this paper is organized as follows. In Section 2, we review related work in saliency prediction. Section 3 and Section 4 introduce the retina model and our proposed saliency prediction framework, respectively. Experimental results on benchmark datasets are presented in Section 5. Finally, Section 6 concludes the paper and outlines directions for future research.

## 2. Related Work

The study of visual saliency has undergone substantial advancements since the seminal work of Itti and Koch [16], who introduced one of the first computational models grounded in feature integration theory. Their model employs a biologically inspired center-surround mechanism, integrating color, intensity, and orientation features across multiple scales to identify salient regions within an image. This foundational approach has inspired a plethora of subsequent models aimed at predicting human eye fixation points.

Building on this foundation, Harel et al. [14] proposed a graph-based saliency model that enhances biological plausibility by generating activation maps for specific feature channels and subsequently normalizing them. This method simplifies the detection of salient areas through an effective yet straightforward mechanism. In a similar vein, Zhang and Sclaroff [17] developed a saliency map generation technique that employs a Boolean combination of binary and attention maps, further refining the identification of salient regions. These early models predominantly leveraged local low-level features, often integrating global statistical information or heuristic combinations of low- and high-level features to improve saliency prediction accuracy.

The introduction of deep neural networks (DNNs) [18] marked a significant leap forward in saliency prediction, markedly enhancing state-of-the-art performance. The eDN model [19] was pioneering in utilizing deep features trained from scratch specifically for saliency prediction. This was followed by DeepGaze I [20], which harnessed DNN features pre-trained on object recognition tasks, resulting in substantial improvements in saliency prediction accuracy. The success of these models underscored the efficacy of transfer learning, capitalizing on the intrinsic relationship between high-level visual tasks, such as object recognition, and human fixation selection. Consequently, subsequent saliency models have largely adopted transfer learning strategies to bolster performance. For example, the SALICON (https://salicon.net/, accessed on 2 March 2025) model [21] fine-tunes a combination of deep features extracted from AlexNet, VGG-16, and GoogLeNet architectures for saliency prediction. Similarly, many state-of-the-art models have focused on fine-tuning VGG-16 features specifically for saliency tasks, highlighting the robustness and versatility of this architecture in capturing salient information.

In our work, we explore the integration of retinal processing models with deep learning-based saliency prediction. Unlike most existing approaches that fine-tune pre-trained VGG-16 networks on original images, our methodology involves preprocessing input images through a retinal model before feeding them into the network. This necessitates training a VGG-16 model from scratch for object recognition, as the input data differ from standard image formats. Additionally, this approach requires training separate models for saliency prediction. Given the limited availability of training code for various models, we construct a DNN for saliency prediction using VGG-16, training it from the ground up and comparing its performance with existing VGG-16-based models. Detailed methodologies and comparative analyses are presented in the following sections.

## 3. Retina Imitation

The human retina is a sophisticated network composed of photoreceptors, horizontal cells, bipolar cells, ganglion cells, and amacrine cells, which collaboratively initiate the complex process of visual information processing [13]. These cellular interactions are organized into two principal layers: the Outer Plexiform Layer (OPL) and the Inner Plexiform Layer (IPL). For the purpose of image saliency prediction, which primarily relies on static visual features rather than motion cues, our focus is directed towards the Parvocellular (Parvo) channel. The Parvo pathway is dedicated to high-resolution detail extraction and efficient information encoding, making it especially pertinent for identifying salient features within an image by reducing entropy and preserving crucial information.

### 3.1. Photoreceptors

Photoreceptors exhibit the remarkable ability to adjust their sensitivity in response to the luminance of their surrounding environment [22]. This adaptive mechanism can be viewed from an information-theoretic perspective as a means to balance the entropy of the received signal, ensuring that salient information is neither lost nor overly redundant. The adaptation is effectively modeled using the Michaelis–Menten relation [23], normalized over a luminance range of $\left[0,V_{\max}\right]$. The adjusted luminance $A_{p}$ of a photoreceptor *p* is mathematically expressed as:

(1) $$\begin{array}{cc} A_{p} & =\frac{R_{p}}{R_{p}+R_{p}^{0}}\cdot V_{\max}+R_{p}^{0} \end{array}$$

(2) $$\begin{array}{cc} R_{p}^{0} & =V_{0}\cdot L_{p}+V_{\max}\left(1-V_{0}\right) \end{array}$$

In these equations, $R_{p}$ denotes the current luminance received by the photoreceptor, while $R_{p}^{0}$ serves as the compression parameter, linearly dependent on the local luminance $L_{p}$ of the photoreceptor’s neighborhood. The parameter $V_{0}$, empirically set to 0.90, fine-tunes the local adaptation effect, balancing sensitivity and precision to enhance the model’s capacity for detailed feature extraction. This sensitivity adjustment effectively reduces the entropy of the input signal, focusing on the most informative aspects of the visual scene.

### 3.2. Outer Plexiform Layer (OPL)

The OPL layer’s cellular interactions are characterized by a nonseparable spatio-temporal filter [22]. The transfer function for a one-dimensional signal within this layer is defined as:

(3) $$\begin{array}{c} F_{OPL}\left(f_{s},f_{t}\right)=F_{ph}\left(f_{s},f_{t}\right)\cdot \left[1-F_{h}\left(f_{s},f_{t}\right)\right] \end{array}$$

**Figure 2:**
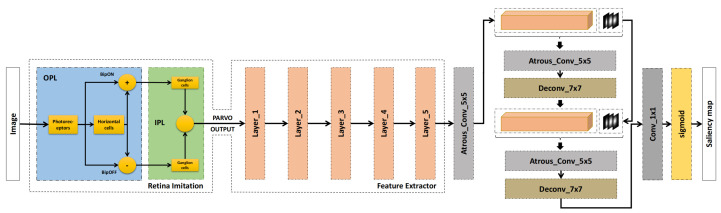
Architecture of a saliency prediction model that combines a retina-inspired module with deep neural networks. The left block (Retina Imitation) simulates early visual processing, mimicking photoreceptors, bipolar cells, and retinal ganglion cells. The output then flows to the middle block (Feature Extractor), which uses multiple deep layers to learn discriminative features. Finally, the right block applies atrous (dilated) convolutions and deconvolution layers to generate the saliency map. This multi-stage design leverages both biological insights and deep learning to more accurately predict human-like visual attention.

Here, $f_{s}$ represents the spatial frequency, and $f_{t}$ denotes the temporal frequency. The functions $F_{ph}$ and $F_{h}$ correspond to the transfer functions of the photoreceptor network and the horizontal cell network, respectively [13]. Essentially, $F_{OPL}$ captures the differential effect between these two networks, modeling the inhibitory and excitatory interactions that facilitate the processing of visual information. From an information theory standpoint, this differential interaction acts to filter out noise and redundancy, thereby reducing entropy and preserving the core information content. As illustrated in Figure 2, this differential is implemented through the BipON and BipOFF operations, which emulate the role of bipolar cells in segregating the OPL outputs into two distinct channels, each carrying maximized informational content.

### 3.3. Inner Plexiform Layer (IPL) and Parvocellular Channel

The outputs from the BipON and BipOFF operations within the OPL are transmitted to the ganglion cells of the Parvocellular channel in the IPL. This transmission is modeled using the Michaelis–Menten law [24], enabling local enhancement of contour information within each channel independently. This enhancement process can be interpreted as an entropy reduction step that reinforces salient contour data, thereby improving the model’s ability to detect and emphasize significant features in the visual input while minimizing redundant information.

In summary, the Parvocellular filtering pipeline integrates photoreceptors, the OPL, and the IPL Parvo models, as depicted in Figure 2. This pipeline adapts to local luminance variations, thereby enhancing fine details in both dark and bright regions of the image and optimizing the information throughput by reducing entropy. In our experiments, we apply the Parvocellular channel processing to each original color image to extract detailed features with a focus on information preservation and entropy minimization. Although this retinal imitation simulates only a subset of the retinal cells, its output effectively isolates image details in a manner analogous to the human retina’s conversion of visual stimuli into spike trains [25]. While spike trains represent the temporal dynamics of neural activity and are inherently complex to interpret, our model produces visually interpretable results and facilitates efficient computation by applying principles from information theory, making it a practical approximation of retinal processing for saliency prediction tasks.

## 4. Exploration of Deep Neural Networks

To develop a computational model that plausibly predicts visual saliency, it is imperative that the model demonstrates high performance in saliency prediction tasks while efficiently managing information flow and entropy. Recent advancements in computer vision, particularly with deep neural networks (DNNs), have revolutionized visual object categorization, achieving unprecedented accuracy [26,27]. The efficacy of DNNs in saliency prediction is further underscored by the success of transfer learning approaches, as exemplified by the DeepGaze I model [20]. This model leverages features from networks pre-trained on object recognition tasks, highlighting the intrinsic connection between high-level visual processing, information representation, and human fixation behavior. Consequently, DNNs emerge as a robust foundation for constructing effective saliency prediction frameworks that inherently manage and reduce entropy through learned feature hierarchies.

### 4.1. Neuroscientific Insights into DNN Representations

The alignment between DNNs and human neural processing has been a subject of extensive research. Cichy et al. [15] demonstrated that neurons in the early layers of DNNs exhibit sensitivities akin to Gabor filters or color patches, reminiscent of the receptive fields observed in the human visual cortex. As one progresses to deeper layers, neurons respond to increasingly complex patterns, paralleling the hierarchical processing in the human brain where higher cortical areas integrate more abstract features. This hierarchical feature representation not only mirrors the spatial and temporal dynamics of human brain activity but also enhances the predictive capability of DNNs in modeling human visual attention. From an information theory perspective, each successive layer of the DNN can be viewed as a transformation that refines the encoded information, reducing entropy and preserving salient features crucial for accurate saliency prediction [15].

### 4.2. Advancements in Network Architectures

Recent architectural innovations in DNNs, such as Residual Networks (ResNet) [26] and Densely Connected Convolutional Networks (DenseNet) [27], have further propelled performance in computer vision tasks. ResNet, with its residual learning framework, enables the training of ultra-deep networks by mitigating the vanishing gradient problem, thereby rivaling human performance on benchmarks like ImageNet. DenseNet, on the other hand, achieves state-of-the-art results with fewer parameters and reduced computational overhead by promoting feature reuse through dense connections. Despite these advancements, a notable distinction between these networks and the human cortex lies in their depth and the nature of their computational processes. Han et al. [28] elucidate that the effectiveness of ultra-deep networks is primarily attributable to their ability to model recurrent computations essential for recognition tasks, a facet that remains underrepresented in current DNN architectures. Additionally, these networks often overlook the temporal evolution of selectivity and invariance characteristic of neural processing in the brain [28]. Viewed through the lens of entropy, these architectures strive to compress and refine high-dimensional data, yet may lack mechanisms to dynamically adjust entropy reduction in a biologically plausible manner.

### 4.3. DNNs as Feature Extractors for Saliency Prediction

In our approach, illustrated in Figure 2, we employ a DNN to extract hierarchical features following retina imitation. The multi-level features, spanning from low-level to high-level abstractions, emulate the information transfer and entropy reduction processes within the human cortex. While our framework accommodates various DNN architectures, such as ResNet, DenseNet, and VGG-16, we opt for VGG-16 due to its widespread use and proven efficacy in feature extraction for saliency tasks [29]. By treating the DNN as a black box, our methodology remains flexible, allowing for the integration of different neural networks to assess their impact on saliency prediction performance and information-theoretic efficiency. However, the predominance of VGG-16 in existing models justifies its selection for a focused examination of retina imitation’s influence on saliency outcomes, particularly in how these networks handle information compression and entropy management.

### 4.4. Incorporating Center Bias

Psychological studies have consistently shown a central bias in human gaze patterns, where observers tend to focus more on the image center [30]. From an information-theoretic viewpoint, this bias implies a higher expected information density at the center of images. To model this phenomenon, we incorporate a set of Gaussian functions with diagonal covariance matrices to represent the center bias [30]. The network learns the parameters of *N* Gaussian functions, generating relative prior maps that introduce non-linearity into the saliency prediction model while encoding spatial priors that reduce uncertainty (entropy) about likely fixation points. Mathematically, the center bias can be represented as:

(4) $$\begin{array}{c} B\left(x,y\right)=\sum_{i=1}^{N}\alpha_{i}\exp\left(-\frac{{\left(x-\mu_{x,i}\right)}^{2}}{2\sigma_{x,i}^{2}}-\frac{{\left(y-\mu_{y,i}\right)}^{2}}{2\sigma_{y,i}^{2}}\right), \end{array}$$

where (x,y) denotes spatial coordinates, $\alpha_{i}$ represents the amplitude, and $\left(\mu_{x,i},\mu_{y,i}\right)$, $\sigma_{x,i}$ are the mean and standard deviation of the *i*-th Gaussian component, respectively. This bias is integrated into the saliency map generation process, enhancing the model’s alignment with natural viewing behaviors by emphasizing regions with higher predicted information content.

Following the application of the center bias, deconvolution operations are employed to progressively align the feature maps with the original input dimensions. Specifically, after each Gaussian bias application, a deconvolution layer (deconv) upscales the feature maps, facilitating the aggregation of multi-scale information while preserving the entropy-reduced salient features. After two such operations, the final saliency map is produced via a 1×1 convolutional layer with a sigmoid activation function, which ensures the saliency values are normalized between 0 and 1.

### 4.5. Model Architecture

The proposed model, termed Retina-VGG16, seamlessly integrates biologically inspired retinal processing with deep feature extraction and center bias modeling. At its core, a pre-trained VGG16 network serves as the feature extractor, converting an input image (of size 224 × 224) into a compact 7 × 7 feature representation. To capture multi-scale contextual information, an Atrous Spatial Pyramid Pooling (ASPP) module is applied to these features. The ASPP module employs parallel 5 × 5 atrous convolutions with varying dilation rates, enabling the model to aggregate information at multiple scales while preserving spatial resolution.

In order to incorporate human visual system biases, a fixed Gaussian center bias map is pre-computed and integrated at multiple stages. At the 7 × 7 resolution, the center bias is resized and combined with the ASPP output via channel-wise concatenation; random weighting is applied to generate multiple bias features. This combined representation is then refined by a 5 × 5 atrous convolution (with dilation rate 2 and appropriate padding), followed by ReLU activation and batch normalization. A learnable upsampling operation, implemented via a PixelShuffle module, increases the spatial resolution from 7 × 7 to 14 × 14 while preserving detailed feature information.

A similar procedure is applied at the 14 × 14 resolution. The upsampled feature maps are again concatenated with resized center bias features (with new random weights) and processed through another 5 × 5 atrous convolution block. A subsequent PixelShuffle upsampling elevates the resolution from 14 × 14 to 28 × 28. Finally, a 1 × 1 convolution reduces the channel dimensionality to 1, and bilinear interpolation is used to scale the output saliency map to the desired size (e.g., 224 × 224). A sigmoid activation function normalizes the final saliency predictions to the range [0, 1].

Mathematically, the saliency map generation can be summarized as:

(5) $$\begin{array}{cc} \mathrm{Saliency}(x,y) & =\sigma \left({\mathrm{Conv}}_{1\times 1}\left(\mathrm{Deconv}\left(\mathrm{Deconv}\left(\mathrm{Feature}\;\mathrm{Map}\times B(x,y)\right)\right)\right)\right), \end{array}$$

where σ denotes the sigmoid function, ${\mathrm{Conv}}_{1\times 1}$, represents the final convolutional layer that maps the refined features to a single-channel saliency map, and B(x,y) is the spatially varying center bias.

To train the network for accurate saliency prediction, a composite loss function is employed that combines the strengths of Focal Loss and Mean Squared Error (MSE) Loss. The Focal Loss is designed to address class imbalance and focus on difficult examples by down-weighting well-classified pixels. It is defined with a focusing parameter γ (set to 2) and a balancing factor α (set to 0.25). In parallel, the MSE Loss ensures pixel-wise similarity between the predicted saliency map and the ground truth, effectively penalizing discrepancies in intensity values. The overall training loss is computed as:

(6) $$\begin{array}{c} L_{\mathrm{total}}=L_{\mathrm{Focal}}+L_{\mathrm{MSE}}, \end{array}$$

where $L_{\mathrm{Focal}}$ emphasizes the hard-to-classify regions and $L_{\mathrm{MSE}}$ enforces global consistency in saliency estimation.

Overall, Retina-VGG16 encapsulates a novel synergy between neurobiological inspiration and modern deep learning techniques. By emulating key aspects of retinal processing—such as photoreceptor adaptation and OPL/IPL filtering—and by explicitly modeling center bias, the architecture achieves efficient information encoding and entropy reduction. This results in saliency maps that are not only more aligned with human fixation patterns but also computationally efficient and theoretically grounded in information theory.

## 5. Experiments

### 5.1. Network Training

To preserve the output size of the network, we modified the VGG-16 architecture by retaining only the first two max-pooling layers. Given an input image resized to 240×320 pixels, the feature maps at each level are downsampled to 60×80 pixels through max-pooling operations, matching the dimensions of the final feature maps. We employ the mean squared error (MSE) as the loss function and set the number of Gaussian function parameters to 16. Since the output saliency map has a lower resolution than the original image, the ground-truth saliency maps are downsampled during training to align with the output dimensions. During inference, the predicted saliency map is upsampled using a bilinear filter to match the input image size, and a sigmoid function is applied to normalize the map into a probability distribution.

We trained the proposed network using the SALICON dataset [31], one of the largest publicly available saliency datasets, containing eye fixation data for 20,000 images. The ground-truth saliency maps were generated by blurring the eye fixation points with a Gaussian kernel of fixed variance [31]. This blurring accounts for noise in eye-tracking equipment and the observer’s saccade landing positions, resulting in saliency maps that are generally smooth without sharp boundaries typical of salient object segmentation ground truths [31]. The dataset encompasses a diverse set of indoor and outdoor scenes with varying levels of clutter. We allocated 10,000 images for training, 5000 for validation, and 5000 for testing. The network was trained with a mini-batch size of eight images per iteration. The entire training process was completed in approximately one day using an NVIDIA TITAN XP GPU (NVIDIA, Santa Clara, CA, USA) with TensorFlow’s DeepLab framework.

### 5.2. Datasets and Evaluation

We evaluated the performance of our proposed model and the enhancement of image saliency through retina imitation on the Toronto [32] and MIT1003 [33] datasets. The MIT1003 dataset comprises 1003 natural indoor and outdoor scenes, while the Toronto dataset consists of 120 color images depicting various indoor and outdoor environments.

For comprehensive performance assessment, we employed a range of commonly used evaluation metrics, including Area Under the Receiver Operating Characteristic Curve (AUC), Earth Mover’s Distance (EMD), Kullback–Leibler divergence (KLdiv), Normalized Scanpath Saliency (NSS), Correlation Coefficient (CC), and similarity. Among these, AUC, available in Borji’s and Judd’s variants, is the most widely used metric, comparing the saliency map against all eye fixation points. The shuffled AUC variant penalizes models that exploit center bias present in eye fixation distributions. EMD measures the dissimilarity by penalizing false positives proportionally to their spatial distance from the ground truth. KLdiv quantifies how one probability distribution diverges from an expected distribution. NSS is sensitive to both false positives and false negatives equally. CC and similarity assess the correlation and similarity between the saliency map and the eye fixation density map, respectively.

### 5.3. Enhancement of Image Saliency Prediction Through Retina Imitation

To investigate the efficacy of retina imitation in enhancing saliency prediction, we evaluated its impact on several established bottom-up saliency detection methods from an information-theoretic perspective. The methods assessed include AIM [32], SIM [34], ICL [35], GBVS [14], SUN [36], and FT [37]. These evaluations were conducted on two prominent datasets: the Toronto dataset [32] and the MIT1003 dataset [33]. By incorporating retina imitation, we aim to reduce the entropy of visual input and enhance the preservation of salient information prior to feature extraction by these methods.

**Figure 3:**
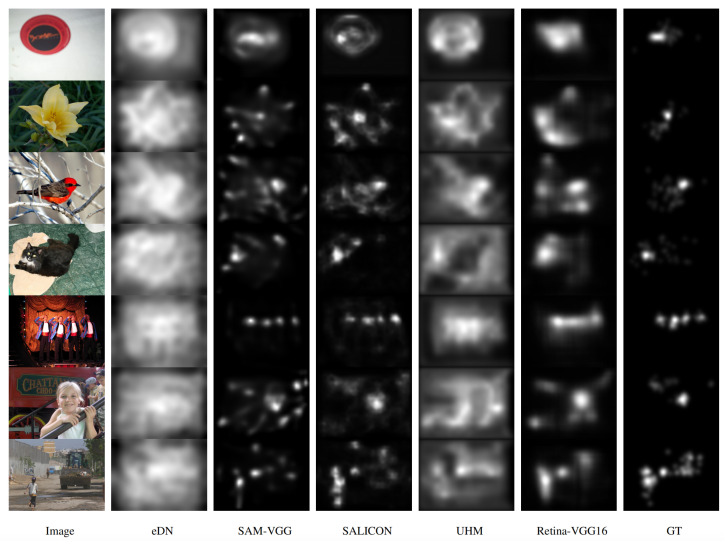
The overall architecture of a saliency prediction model that integrates a biologically inspired retina module with deep neural networks. This multi-stage design leverages both biological plausibility and deep learning capabilities to more accurately predict human-like visual attention.

Table 1 presents a comparative analysis of saliency maps generated from the original images versus those processed with retina imitation across the aforementioned methods. The evaluation employed a comprehensive set of metrics, including Area Under the Receiver Operating Characteristic Curve (AUC), Earth Mover’s Distance (EMD), Kullback–Leibler divergence (KLdiv), Normalized Scanpath Saliency (NSS), Correlation Coefficient (CC), and similarity measures. Metrics such as KLdiv are inherently information-theoretic, measuring the divergence between predicted and actual fixation distributions, thus directly reflecting changes in entropy. As illustrated in Figure 3, approximately 87% of the evaluated cases demonstrated improvements in saliency prediction when retina imitation was applied, indicating more efficient information encoding and entropy reduction in the predicted maps.

Notably, metrics such as similarity, KLdiv, and AUC_Borji_ exhibited substantial enhancements across all bottom-up methods within the Toronto dataset. This indicates that retina imitation effectively aligns the predicted saliency maps more closely with the ground truth eye fixation data by optimizing the information content and reducing redundancy. However, the degree of improvement observed in the MIT1003 dataset was comparatively modest, suggesting dataset-specific information dynamics. Despite this, retina imitation consistently contributed to the overall enhancement of saliency prediction performance across both datasets by reducing unnecessary entropy and focusing on high-information regions.

Furthermore, we benchmarked our proposed model against state-of-the-art saliency prediction frameworks, including UHM [38], SALICON [21], SAM-VGG [30], and eDN [19], as shown in Table 2. Most of these models leverage the VGG-16 architecture for feature extraction and implicitly manage information flow through learned representations. In contrast, our model was trained from scratch, without reliance on pre-trained networks, emphasizing the role of explicit retina imitation in entropy management from the outset. The results indicate that our model achieves either the best or second-best performance across most evaluation metrics on both the Toronto and MIT1003 datasets, with the exception of AUC_Borji_, illustrating its robustness in effectively reducing informational entropy and enhancing salient feature detection.

These findings underscore the effectiveness of retina imitation as a preprocessing step in saliency prediction pipelines from an information theory standpoint. By emulating the human retina, our approach reduces entropy and enhances the capability of existing saliency models to produce outputs that are more congruent with human eye fixation patterns. Consequently, retina imitation represents a valuable augmentation for improving the accuracy, reliability, and information efficiency of saliency prediction systems.

## 6. Conclusions

In this study, we explored the enhancement of image saliency prediction through the imitation of the human visual system’s (HVS) retinal processes, underpinned by principles of entropy and information theory. We introduced a novel saliency prediction framework that integrates retinal imitation—specifically focusing on the Parvocellular channel—to mimic the HVS’s capability for detailed feature extraction and efficient information encoding. Our comprehensive experiments across multiple benchmark datasets demonstrate that applying retina imitation to original images consistently improves the performance of various saliency prediction models by reducing entropy and preserving critical information.

The proposed framework not only outperforms several state-of-the-art methods but also aligns more closely with the biological mechanisms of the HVS. By simulating retinal processes, our approach effectively enhances local detail extraction, mitigates background clutter, and optimizes information flow, leading to more accurate and reliable saliency maps that better correspond to human eye fixation patterns. These improvements underscore the potential of incorporating neuro-inspired preprocessing techniques and information-theoretic strategies in computational models to advance the field of visual saliency prediction through entropy reduction and efficient data representation.

Furthermore, our Retina-VGG16 model, which combines retinal imitation with deep neural network feature extraction, achieved superior performance compared to traditional bottom-up and deep learning-based saliency models. This synergy between biologically inspired mechanisms and advanced machine learning architectures highlights the importance of interdisciplinary approaches in developing more effective and biologically plausible computational models that manage and reduce informational entropy across processing stages.

Looking forward, we aim to extend our framework by incorporating the Magnocellular (Magno) channel, which is dedicated to motion information extraction in the retina. Integrating the Magno channel will enable our model to account for dynamic visual cues, further enhancing saliency prediction in scenarios involving motion by dynamically adjusting entropy in response to temporal changes. Additionally, future work will explore adaptive retina imitation techniques tailored to specific dataset characteristics, as well as the integration of other HVS components, to achieve a more comprehensive simulation of human visual processing and more efficient information encoding.

In conclusion, our research demonstrates that biologically inspired retinal imitation, grounded in entropy and information theory, significantly enhances saliency prediction models. This advancement paves the way for more accurate and human-aligned computational vision systems, contributing to the theoretical understanding of visual saliency mechanisms while offering practical implications for applications such as image compression, object detection, and human–computer interaction through optimized information processing and entropy management.

## Figures and Tables

**Table 1:** Comparison results of the original image and the image applied retina imitation on the Toronto dataset [32] and the MIT1003 dataset [33]. Most of the methods listed in this table belong to the bottom-up saliency prediction models. Scores in bold mean the better performance between the original image and the image applied retina imitation.

Metrics	CC		similarity		EMD		KLdiv		NSS		${\mathrm{AUC}}_{\mathrm{Borji}}$		${\mathrm{AUC}}_{\mathrm{Judd}}$		${\mathrm{AUC}}_{\mathrm{shuffled}}$	
**Baseline**	**1**		1		**0**		**0**		**3.29**		**0.88**		**0.92**		**0.81**	
**Types**	**Image**	**Retina**	**Image**	**Retina**	**Image**	**Retina**	**Image**	**Retina**	**Image**	**Retina**	**Image**	**Retina**	**Image**	**Retina**	**Image**	**Retina**
**Toronto [32]**																
AIM [32]	0.31	**0.32**	0.38	**0.39**	3.45	3.49	1.36	**1.34**	0.90	0.89	0.72	0.72	0.73	0.73	0.63	0.63
SIM [34]	0.35	0.35	0.38	0.38	3.66	3.68	1.22	1.21	0.96	0.96	0.74	0.74	0.75	0.75	0.66	0.66
ICL [35]	0.45	0.45	0.44	**0.46**	2.94	**2.90**	1.07	**1.01**	1.28	1.30	0.75	0.76	0.79	0.79	0.66	0.66
GBVS [14]	0.57	0.57	0.49	**0.50**	2.30	2.30	0.85	**0.83**	1.52	1.53	0.82	0.82	0.83	0.84	0.70	0.70
SUN [36]	0.22	**0.24**	0.35	0.36	3.70	**3.67**	1.43	1.35	0.64	0.71	0.66	0.68	0.67	0.69	0.59	0.61
FT [37]	0.10	**0.26**	0.31	0.37	4.22	**3.78**	1.65	**1.41**	0.30	0.75	0.58	0.67	0.59	0.69	0.55	0.61
**MIT1003 [33]**																
AIM [32]	0.23	0.22	0.28	0.27	5.94	6.02	1.79	1.81	0.78	0.81	0.70	0.70	0.71	0.71	0.59	0.59
SIM [34]	0.23	0.23	0.27	0.27	6.18	6.25	1.73	1.72	0.78	0.79	0.70	0.70	0.71	0.71	0.59	0.60
ICL [35]	0.32	0.32	0.34	0.34	5.06	5.06	1.50	1.48	1.09	1.09	0.73	0.74	0.77	0.77	0.60	0.60
GBVS [14]	0.42	0.42	0.36	0.36	4.34	4.36	1.30	1.30	1.37	1.36	0.81	0.81	0.82	0.82	0.63	0.63
SUN [36]	0.19	0.19	0.26	0.26	6.12	6.16	1.89	1.86	0.66	0.66	0.66	0.66	0.67	0.67	0.57	0.57
FT [37]	0.05	0.09	0.21	0.22	6.65	6.31	3.01	3.61	0.19	0.33	0.53	0.54	0.57	0.57	0.51	0.52

**Table 2:** Comparison results of UHM [38], SALICON [21], SAM-VGG [30], eDN [19], and Reina-VGG16 on the Toronto dataset [32] and the MIT1003 dataset [33]. Scores in bold represent the best performance.

Metrics	CC	Similarity	EMD	KLdiv	NSS	${\mathrm{AUC}}_{\mathrm{Borji}}$	${\mathrm{AUC}}_{\mathrm{Judd}}$	${\mathrm{AUC}}_{\mathrm{shuffled}}$
**Toronto [32]**								
**UHM [38]**	0.57	0.50	2.30	0.83	1.53	0.82	0.84	0.70
**SALICON [21]**	0.56	0.48	2.35	0.87	1.47	0.82	0.83	0.70
**SAM-VGG [30]**	0.57	0.49	2.30	0.85	1.51	0.82	0.84	0.70
**eDN [19]**	0.50	0.40	3.06	1.12	1.25	**0.84**	**0.85**	0.71
**FixPNet-E2 [39]**	0.54	0.33	7.09	1.42	1.26	0.79	0.81	0.69
**Reina-VGG16**	**0.64**	**0.56**	**1.96**	**0.68**	**1.73**	0.82	**0.85**	**0.72**
**MIT1003 [33]**								
**UHM [38]**	0.42	0.33	4.99	1.41	1.35	0.81	0.82	0.63
**SALICON [21]**	0.52	0.41	4.21	1.14	**1.92**	0.78	0.83	0.67
**SAM-VGG [30]**	0.49	0.42	3.81	1.28	1.74	0.77	**0.85**	0.66
**eDN [19]**	0.41	0.30	5.32	1.55	1.29	**0.85**	**0.85**	0.66
**FixPNet-E2 [39]**	0.48	0.24	8.00	1.86	1.29	0.82	0.83	0.66
**Reina-VGG16**	**0.54**	**0.44**	**3.76**	**1.03**	1.82	0.84	**0.86**	**0.68**

## Data Availability

All the relevant data generated in this study have been deposited in the repository under https://github.com/iCGY96/Retina-VGG, accessed on 2 March 2025.
